# Diphtheria, Bacteria, and Dr. Gregg

**Published:** 1881-11

**Authors:** P. P. Wells

**Affiliations:** Brooklyn, N. Y.


### DIPHTHERIA, BACTERIA, AND DR. GREGG.

P. P. WELLS, M. D., BROOKLYN, N. Y.

In the June number of THE HOMŒOPATHIC PHYSICIAN, I took occasion to protest against the antiquated sin of giving hypothesis for fact in medical writing or teaching. This was suggested by eight pages of printed matter to which the name of Dr. Gregg was attached as author, and which, so far as I could see then, or can see now, was composed so exclusively of imaginary elements, as to afford good opportunity for the protest, if indeed it did not impose this as a duty.* Dr. Gregg objects to my protest, and attempts to give an answer to it. With what success he accomplishes this we shall soon see.

The points of the eight pages which I criticised were:

1st. The exudations in diphtheria * * * are fibrin.

2d. Fibrin is always in excess in the blood in diphtheria.

---

* In his reply to my critique Dr. Gregg objects that I omitted to notice matters, notably proofs, which were in a journal before me, and he assumes that it was what he had there published that I was engaged with. This is a mistake. The eight pages I criticised were in pamphlet form, and received by a professional neighbor, and as he supposed from Dr. Gregg himself. My neighbor handed them to me with the suggestion that I should review them. It was supposed by my neighbor and myself, that, coming from the author, he was willing to stand and be judged by them, as they had come to our hand. The eight points I criticised were there, as naked of every thing like proof of the truth of these hypotheses, as truth herself is, traditionally, of all dry-goods from dress-making hands. Hence I called them *hypotheses*, which the Doctor says is "*false.*" See Worcester's Unabridged, word "*Hypothesis.*"

3d. This excess is a fact in all other inflammatory diseases.

4th. That excess of fibrin is always a source of more or less danger in pleuritis, peritonitis, etc.

5th. This excess of fibrin is disposed to roll itself into clots, and plug up either the heart or arteries, and so cause death.

6th. The specific exudation in diphtheria is a beneficent effort of nature to relieve the heart and arteries of danger from the excess of fibrin in the blood.

7th. Every case of diphtheria would prove fatal if this excess were not so expelled from the circulation.

8th. Bacteria, or that which has been so called, is only fibrin.

The above are the points in Dr. Gregg's paper I criticised. I intended to do this fairly. I still believe my performance justifies my intention. My chief object was to endeavor to put *hypothesis* in scientific matters in its true place, and this performance of Dr. Gregg, in carrying out this object, was used to illustrate its general worthlessness. In his critique of my criticism, after a few personal remarks which require no other notice than an expression of thankfulness for his good opinion, he takes exception to my use of the word *hypothesis*, and declares "it is false as applied to my inves-it gations and conclusions, in any department of pathology," etc. I still believe the word fitly applies to each of the eight particulars in the pamphlet which I dealt with, and now, after reading his attempted reply, know no other word which so exactly characterizes them. They seem to me hypotheses, pure and simple. I had to do with these eight, each of them as he had made them, relating to diphtheria, and with no other of his "investigations," or "conclusions" in other matters distinct from this. His first attempt to convince his critic that he had "builded securely and permanently," presumably as to this matter of diphtheria, as I have had no connection with aught else of his labors, is to enter a declaration as to his "investigations and conclusions" as to *phthisis*, a matter with which I had in no manner meddled, and which, so far as I can see now, has no more to do with these eight points criticised, than it had with the orbit of the last telescopic comet. I would not in the least detract from any merit pertaining to the Doctor's long labors and discoveries, if he has made any. But I do not see how twenty years spent in the study of *phthisis* can change the hypothetic character of either of the criticised points, these having only to do with a matter wholly distinct from this; viz. *diphtheria*. But the Doctor claims to have given almost equal time and labor to the study of this last, as to tubercle, and it would seem that he would therefore claim for these eight points that this long labor should raise them above the level of hypothesis in some way or other. As they stand in the criticised pamphlet, wholly unsustained by any of the facts gathered in this long study, I do not see how this can be. They appear there as "a *theory founded on a principle unproved.*"

The Doctor mistakes in supposing his paper, the subject of my criticism, was found in a "journal." What he had said in a journal of the impracticability of giving "one-twentieth of the proof" in existence "to sustain the views here presented" I have had no means of knowing, but what we do know is, that in the paper I criticised there was just no proof at all, and the whole matter rested, so far as that paper is concerned, on the dictum of the Doctor alone, just as I said. I repeat this, though he meets my statements with some bitterness. There is not a trace of proof given that I remember. It was this which left the eight points mere hypotheses. The Doctor assures us he "makes no statements without the best authority, or the best reasons for making them." This may be all just so, but what is wanted to give these eight points the least value, is not "*reasons*" nor "authority," if by this he means other men's names, but the *facts* on which they rest their claim to our respect. In the pamphlet criticised is not one such found. I am not concerned just now with what the Doctor is, or is not "accustomed" to do. But solely with what he *has done* or has not done in the pamphlet criticised.

As to the matter of bacteria, I have never attached much importance to them any way, and none whatever, as I have said, to them as a cause of which diphtheria is the effect. But when it is a question whether I shall receive the testimony of Professor Hauft, in a matter which he has seen and investigated, or that of Dr. Gregg as to the same matter, he not having seen it, I should certainly give the professor my credence every time.

The imagination of the Doctor seems to be capable of other extraordinary feats than those expended on his supposed facts of diphtheria. "Do you not see," he says, "in this criticism, written ostensibly to condemn all pathological investigations," etc. How ostensibly? I have not mentioned pathological investigations, in whole or in part. And "you have placed yourself in the unenviable position of an eminent advocate of a false pathology," etc. I have not, to my knowledge, advocated any pathology, true or false, earnestly or otherwise, in the matter of diphtheria, and therefore do not see that I have placed myself in the position he suggests. But the pathology he will have it I am advocating, he says is not only false, but perniciously false at that.* Well, well, here is a new exhibition of the power of imagination. It is up fully to the Hudibrastic standard—

"Optics sharp it takes, I ween,
To see what is not to be seen."

Then, the Doctor insists upon it, I tried to "ridicule nature." Don't make a mistake, Doctor. It was only the ridiculous service you had imposed upon the worthy matron. Not the lady herself, but the hypothetical function your imagination had imposed on her. Do you see the difference?

As to the question of the neccessary fatality in all cases of diphtheria which lack the membrane, the Doctor simply begs the question and then proceeds to argue the matter as if the question had been proved. This is not a new dodge in logic, nor is it one held in much respect by the learned world.

Now, would it be thought possible, that after these accusations of offences I have not committed, and boastings of having given more years to the study of this subject than I have weeks, and informing me that my "enmity to pathology" has "betrayed me into a neglect of pathological reading,"† the Doctor would generously propose that I shall meet him in a public discussion of this subject. Of course he did not expect his proposition would be accepted. He ought to have known that everybody, except the writer of these eight criticised points, would at once say there can be no gain to anybody or to any subject by debate with one who fails to distinguish assertion from proof, and who has

---

*But the Doctor makes me out even worse than this. If we may take his word for it, I am "a defender of and apologist for an allopathic and mongrel pathology of diphtheria, that is reeking through and through with fallacies, and which has led to much worse treatment than could otherwise have been thought of." And then he says he is "astonished." No wonder. So am I. I didn't know I was so bad, and am the more astonished because, so far as I remember, I have never said a word on the subject of this pathology, except to dissent from that of these eight points, if this may be so construed. I did not regard that as equal to a conviction of the seven mortal sins. Am I mistaken?

† So it is. I wrote my criticism to show my "enmity" to "all pathological investigations by our school," and more than this, the same "enmity" "has betrayed you [me] into a great neglect of pathological reading." Now the such a facility for accusation of his opponent as to cease all discrimination between what he has said and what he has not, and no less facility in ascribing to him enmities and partialities of which he is neither guilty nor capable, and an equal facility to foist them into the importance of argument, all, of course, on his side. There can be no gain in debating with such a man, any subject whatever. And I may add, if the outcome of his twenty years' study of this subject is contained in the eight pages I have criticised, then, I can say the long labor was very like to that of the poet's mountain, the product of which was only a "*ridiculus mus.*"

